# Using formative research to develop *CHANGE!*: a curriculum-based physical activity promoting intervention

**DOI:** 10.1186/1471-2458-11-831

**Published:** 2011-10-27

**Authors:** Kelly A Mackintosh, Zoe R Knowles, Nicola D Ridgers, Stuart J Fairclough

**Affiliations:** 1 Faculty of Education, Community and Leisure, Liverpool John Moores University, Barkhill Road, Liverpool, L17 6BD, UK; 2 REACH Group, Liverpool John Moores University, Liverpool, Byrom Street, L3 3AF, UK; 3 Research Institute for Sport and Exercise Sciences, Liverpool John Moores University, Liverpool, Byrom Street, L3 3AF, UK; 4 Centre for Physical Activity and Nutrition Research, Deakin University, Burwood Victoria 3125, Australia

## Abstract

**Background:**

Low childhood physical activity levels are currently one of the most pressing public health concerns. Numerous school-based physical activity interventions have been conducted with varied success. Identifying effective child-based physical activity interventions are warranted. The purpose of this formative study was to elicit subjective views of children, their parents, and teachers about physical activity to inform the design of the CHANGE! (Children's Health, Activity, and Nutrition: Get Educated!) intervention programme.

**Methods:**

Semi-structured mixed-gender interviews (group and individual) were conducted in 11 primary schools, stratified by socioeconomic status, with 60 children aged 9-10 years (24 boys, 36 girls), 33 parents (4 male, 29 female) and 10 teachers (4 male, 6 female). Questions for interviews were structured around the PRECEDE stage of the PRECEDE-PROCEDE model and addressed knowledge, attitudes and beliefs towards physical activity, as well as views on barriers to participation. All data were transcribed verbatim. Pen profiles were constructed from the transcripts in a deductive manner using the Youth Physical Activity Promotion Model framework. The profiles represented analysis outcomes via a diagram of key emergent themes.

**Results:**

Analyses revealed an understanding of the relationship between physical activity and health, although some children had limited understanding of what constitutes physical activity. Views elicited by children and parents were generally consistent. Fun, enjoyment and social support were important predictors of physical activity participation, though several barriers such as lack of parental support were identified across all group interviews. The perception of family invested time was positively linked to physical activity engagement.

**Conclusions:**

Families have a powerful and important role in promoting health-enhancing behaviours. Involvement of parents and the whole family is a strategy that could be significant to increase children's physical activity levels. Addressing various perceived barriers to such behaviours therefore, remains imperative.

**Trial Registration:**

ISRCTN: ISRCTN03863885

## Background

Numerous physiological health and psychological well-being benefits associated with regular physical activity have been documented [1,2]. Current United Kingdom (UK) and international physical activity guidelines recommend that children undertake health-enhancing moderate-to-vigorous physical activity (MVPA) for at least 60 minutes over the course of each day [3,4]. Despite this, low levels of children's physical activity are commonly reported, with a recent large scale study observing that only 5.1% of boys and 0.4% of girls met current recommendations when measured using accelerometry [5].

Although the prevalence of childhood obesity is thought to have 'levelled-off' in recent years, previous stable phases have been followed by further increases, and the current prevalence of obesity remains extremely high [6]. Reversing the prevalence of childhood overweight and obesity, therefore, is still an important public health priority, since childhood obesity tracks through adolescence [7] and into adulthood [8], and increases the risk of adult premature mortality [9]. Numerous strategies and school-based interventions to tackle obesity through enhanced physical activity have been implemented, though few studies have demonstrated sustained behavioural change (i.e., more than one year) or positive impacts on children's health and well-being [10].

Despite large-scale quantitative studies being able to assess the direction and strength of trends in participation of physical activity, they are unable to explain the reasons why children and significant others (i.e., parents and teachers) maintain or cease to participate in life-long physical activity [11]. Intervention and strategy development, therefore, have largely overlooked the views of potential participants [12] even though, according to Potvin et al. [10], the need to consult and engage intervention participants (e.g., children, parents, and teachers) within the context of their community has been advocated for some time. Furthermore, a comprehensive understanding of the perceived benefits and barriers to physical activity, afforded by qualitative research, is deemed imperative in the design of successful interventions [11,13]. Such approaches have effectively informed previous physical activity interventions (e.g., [14-16]). Although focus group studies have examined physical activity correlates in children, there is a paucity of research directly comparing the views of children, parents and teachers on the issues relevant to any proposed intervention [17]. There is therefore a need to use informed methods based on extracting such views to design and develop a school-based physical activity intervention, which aim to encourage health-promoting behaviour change.

Behaviour change is complex to both achieve and maintain. In order to develop a successful physical activity-based intervention, an appropriate conceptual health promotion model should be utilised to prioritise the key assets of the target group [13]. One such model is PRECEDE-PROCEED [18], which provides the target population with a comprehensive and structured assessment of their own needs and barriers to a healthy lifestyle. When applied to a tailored intervention programme it is suggested that this approach promotes successful and sustained participant compliance to the intervention protocol [19]. Effective physical activity promotion strategies and interventions are based on known correlates of youth physical activity [20,21], and increases in physical activity have been linked to a range of social, behavioural, physical and social environmental correlates [22]. Inter-relationships between these correlates have been proposed the Youth Physical Activity Promotion Model (YPAPM) [23], which is based on the PRECEDE-PROCEED health promotion model [18]. This hierarchical model is specifically relevant to children's physical activity, and has been previously used in correlates research [24]. The model is underpinned by four categories of correlates termed personal demographic, predisposing, enabling, and reinforcing factors.

Whilst research has generally documented children's physical activity levels, there are less comprehensive data examining underlying reasons and choices for different behaviours. Moreover, research into antecedents and determinants of regular physical activity has predominantly used quantitative methods to identify cross-sectional views to individual's knowledge, attitudes and beliefs towards physical activity in predetermined categories [20]. The aims of this study were to (i) elicit the views of primary (called elementary internationally) school children aged 9-10 years old, their parents, and teachers in relation to their own knowledge, behaviours and perceptions towards childhood physical activity, and to examine perceived benefits and barriers to participation; and (ii) use these data to subsequently inform the design of a tailored physical activity intervention programme, *CHANGE! *(Children's Health, Activity, and Nutrition: Get Educated!).

## Methods

### Participants

Fourteen schools across a large north-west England Borough, with a population of approximately 300,000, were invited to participate in the study and 11agreed to take part (78.6% response rate). The schools were clustered within five pre-defined geographical areas known as Neighbourhood Management Areas (NMA), and stratified by the percentage of students per school eligible to receive free school meals, which was used as a measure of school-level socioeconomic status (SES). One high and one low SES school per NMA were randomly selected to take part to ensure representation of the diverse geographical and social contexts present within the locale. In one NMA two high SES schools were included due to the withdrawal and subsequent late re-inclusion of one school into the study. The children were all white British, which was representative of the dominant ethnic background of the children within the town.

Three hundred and twenty five children in consenting schools were eligible to take part and 203 provided informed written parental consent and child assent (63% participation rate). For the purpose of this formative study a sub-sample of children from each school were randomly selected, stratified by gender, using a random number generator, to provide a representative sample for the population-based approach for the CHANGE! intervention. Consenting and available parents and Year 5 teachers were asked to participate in group interviews and interviews, respectively. Sixty Year 5 children (aged 9-10 years, 24 boys, 36 girls), 33 parents (4 male, 29 female), and 10 teachers (4 male, 6 female) participated in the project. Ethical approval was granted by Liverpool John Moores University Ethics Committee.

### Procedures

The first author facilitated separate semi-structured group interviews involving 3-5 child participants (13 group interviews, n = 60), and 3-8 parent participants (9 group interviews, n = 33). Group interviews with children are deemed a viable method for exploring perspectives if groups are small in composite number [25]. Further, smaller group interview sizes have been recommended for research with children as opposed to adults [26,27], and the range of participants in the majority of our group interviews (4-5) has shown to be optimal in generating good-quality data from children [27]. Previous qualitative physical activity research reported that group interviews with less than 6 participants were conducted successfully [28]. For pragmatic reasons eight interviews were also conducted with 10 teachers, whereby both teachers from two-form entry schools were included in the interview. All interviews utilised the PRECEDE stage of the PRECEDE-PROCEDE model [18] within its design. Across the various interviews, questions were designed appropriately for the format and age of the participants to address knowledge, attitudes and beliefs towards child health and physical activity, as well as views on families' physical activities and barriers to participation. Sample questions from the interviews are presented in Table 1. These questions demonstrated aspects of face validity. The second author, an expert in the field, provided feedback as a Chartered Psychologist. As an example of this protocol, for the children's group interviews the facilitator sat on the floor in a circle with the children to put them at ease and used prompt cards to accommodate children's differing levels of competence, comprehension and attention spans [25]. Both group interviews and individual interviews took place in an appropriate quiet area within school, and lasted 30-45 (mean = 35.2) minutes. All group and individual interviews were recorded using a digital recorder and video recorder, and were transcribed verbatim for further analysis. In total, 30 interviews/group interviews were conducted resulting in 426 pages (228, 122 and 76 pages for children, parents and teachers, respectively) of raw transcription data.

**Table 1:** Example Interview Questions

Interview	Topic	Examples
Children's	Health	What do you think health means?
		What do you think you can do to stay healthy?
Children's	Physical Activity	Who can tell me what physical activity is?
		What things stop you from doing physical activity?
Parents	Health	What can you do to help children be healthy?
		What things to you think could prevent children from being healthy?
Parents	Physical Activity	What things do you think could help your child be physically active?
		Describe any physical activities you do regularly as a family.
Teachers	Health	What things can help children lead healthy lifestyles?
Teachers	Physical Activity	What things do you think could help children be physically active?

### Data analysis

Recent methodological debate in the health literature has discussed the contribution of qualitative studies to the advancement of understanding children's physical activity behaviours [11]. Several authors in exercise related fields have stated the need for different methodologies within qualitative research and 'creativity and flexibility' within analysis procedures [29-31]. Despite various analytic approaches being undertaken, such as manual tagging, 'cut and paste' using word processing data files, or specialist qualitative data analysis packages, such as NVivo, none of these approaches have been shown to directly influence the validity of the study [32]. Recent research in children's physical activity has adopted a pen profile approach [31]. In supporting new methodologies and data representation within qualitative research, pen profiles were constructed from the transcripts of the group interviews and interviews using a manual protocol [31,33]. Pen profiles are considered appropriate for representing analysis outcomes from large data sets via a diagram of composite key emergent themes. This technique presents findings in a manner that is accessible to researchers who have an affinity with both quantitative and qualitative backgrounds [33]. As akin to more traditional group interview data analysis verbatim quotations were then used directly from the transcripts in order to expand the pen profiles.

Methodological rigour was demonstrated using 'trustworthiness criteria' (e.g., [31,34]), whereby the primary researcher deliberated with the other authors that the findings were worthy of attention [35]. The pen profiles and verbatim quotations were initially presented by the first author to the research group, by means of co-operative triangulation. These authors critically questioned the analysis and cross-examined the data in reverse, from the pen profiles to the transcripts. This process was repeated, allowing the authors to offer alternative interpretations of the data, until an acceptable consensus had been reached. Verbatim transcription of data and triangular consensus procedures afforded credibility and transferability, with comparison of pen profiles with verbatim citations accentuating dependability.

## Results

### Pen profiles

Data were initially analysed through a deductive process using the YPAPM [23] as a thematic framework which reflects the underlying study objectives. An inductive process also enabled additional or emergent themes to be further explored [36]. Children's and adults' (both parents and teachers) data are presented independently and structured towards the elements of the YPAPM [23], and in relation to children's physical activity knowledge and barriers. Data were categorized, with personal demographic factors (i.e., SES and gender) explored throughout, rather than independently presented.

### Predisposing Factors

**Figure 1:**
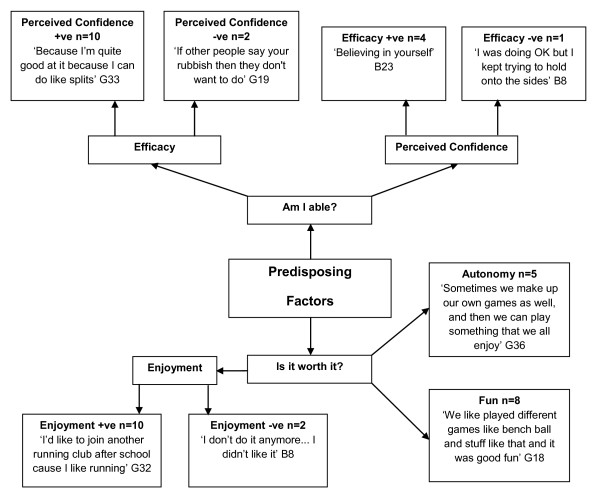
**Children's Predisposing Factors**. +ve = positive. -ve = negative. B = Boy. G = Girl.

Data revealed that children tended to participate in physical activity if they perceived themselves to be physically able (i.e., had the skills), and if they felt it was worthwhile (Figure 1). Those who had high perceived confidence (n = 10) and self-efficacy (n = 4) reported a high level of physical activity participation, contrary to those who had low levels of confidence (n = 2) and self-efficacy (n = 1). A range of physical activities participated in by the children were discussed, with the most common being organised sports (i.e., football, rugby and gymnastics), bike riding, swimming, trampolining, and walking. Though the activities undertaken varied across the cohort, a consistent reason for physical activity participation was to have fun (n = 8) or for enjoyment (n = 10). Several children reported playing specific games they had 'made up'. For example, one child stated that:

"...sometimes we make up our own games as well and then we can play something that we all enjoy playing." (G36)

All children expressing a sense of choice, such as devising a playground game, were from low SES backgrounds, yet the majority of those who specifically mentioned enjoyment (80%) and fun (75%) were from high SES backgrounds.

**Figure 2:**
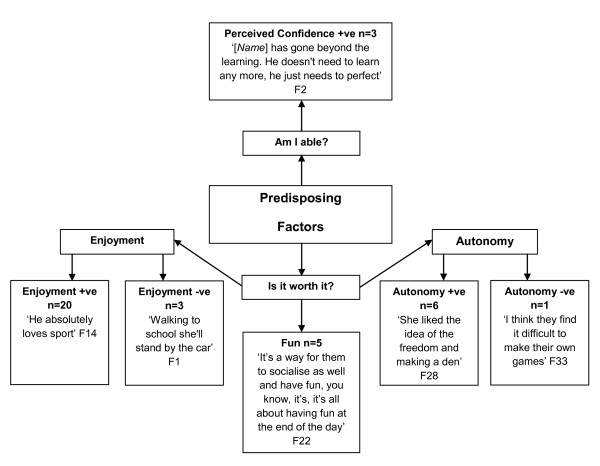
**Adults' Perceived Predisposing Factors to Children's Physical Activity**. +ve = positive. -ve = negative. F = Female.

Data on adults' experiences and perceptions of children's physical activity (Figure 2) indicated that parent's and teacher's consider fun and enjoyment to impact either positively (n = 25), or negatively (n = 3) (i.e., walking to school) on children's physical activity levels: "walking to school she'll stand by the car and I'll have to walk down the street before she'll come after me". Similarly, parents and teachers stated that children's creativity and inventiveness positively influenced their physical activity participation. In addition, teachers (67%) highlighted the relationship between children's perceived confidence and sustained physical activity, as well as encouraging others:

"...the children who have got talent will mix with all abilities...they will try and improve [*the other children*]..." (F32)

### Barriers

**Figure 3:**
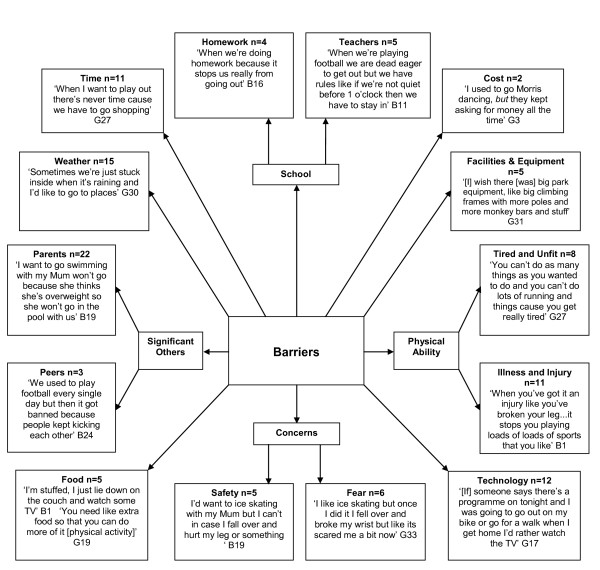
**Barriers to Children's Physical Activity**. B = Boy. G = Girl.

A range of barriers were suggested by the children in relation to physical activity participation both at home and within school time (Figure 3). Similar barriers were reported by children from low and high SES schools, although children from one low SES school quoted cost (financial) as a barrier and didn't perceive safety to be an issue. Children from high SES schools reported dropping out of physical activity more so than peers attending low SES schools. This was often due to fears about risk of physical injury from participation. Not only did children from high SES schools reveal more cases of fear (83%), but illness or injury (82%), time (91%) and the weather (73%) were also reported as barriers to physical activity participation. The data suggest that children perceived their parents as the biggest barriers to their physical activity participation (37%), regardless of SES or gender. Reasons for this include parental social physique anxiety (i.e., not taking their child swimming due to parents' body dissatisfaction), 'grounding' children as a form of punishment, and instructing children to 'stop running around'. In contrast, fewer children talked about peers delimiting engagement in physical activity.

**Figure 4:**
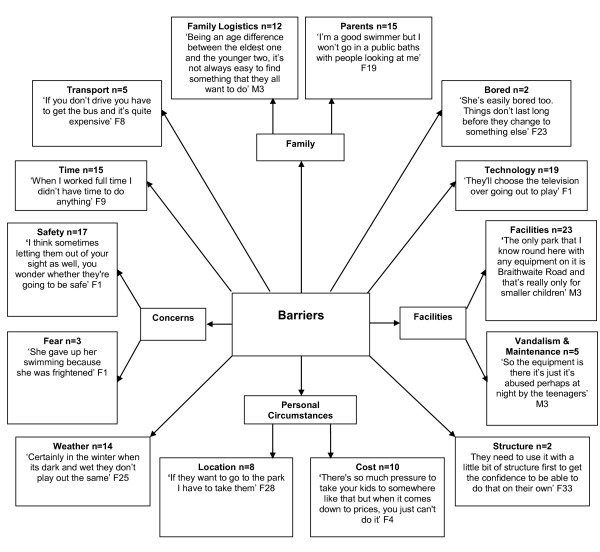
**Adults' Perceived Barriers to Children's Physical Activity**. F = Female. M = Male.

Adults also identified a large range of barriers (Figure 4). Similar barriers were reported between parents and teachers, with the majority relating to parental constraints that were closely linked to time (n = 15), cost (n = 10), safety (n = 17), and family logistics (n = 12), such as a large age gap between siblings limiting the range of feasible family activities. Indeed, some parents from higher socioeconomic areas stated that their children were not allowed to go to the local park, for example, without adult supervision. Such children were therefore reliant on their families for such activity. Teachers (n = 2) suggested lack of structure compromising participation in physical activity. Parents (n = 19) indicated that the advancement in screen-based media has a part to play in decreasing levels of physical activity, yet this was not mentioned by teachers. Moreover, parents themselves seem to play a part in restricting their children's physical activity (n = 15), although this was deduced from the data so the majority of parents were not aware of this.

### Reinforcing factors

**Figure 5:**
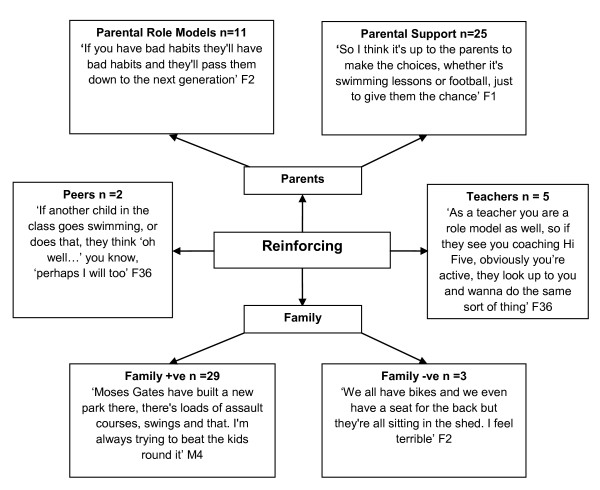
**Adults' Perceived Reinforcing Factors to Children's Physical Activity**. B = Boy. G = Girl.

Key emergent themes (Figure 5) identified by adults were the role of family (n = 29) and parents (n = 25) in supporting and acting as role models (n = 11). One parent described how they had to persevere with taking their child swimming as their child thought they were going to choke, however, simply splashing around with the family without pressure led to their child enjoying it. In addition, teacher views agree with those of parents and demonstrated an appreciation of the influence of their support and peer support.

Children identified a significant need for family support (n = 50), as well as parental (n = 25), peer (n = 23), and teacher/coach (n = 12) support for physical activity participation. However, children from more deprived backgrounds felt less need to have parental (28%) and teacher (17%) support than peers from higher SES schools (Additional file 1, Figure S1).

### Enabling factors

Almost all children (97%) identified having access to facilities and equipment as enabling their participation in physical activity (Additional file 2, Figure S2). Weather emerged as an enabling factor (n = 17), in addition to having dogs (n = 11), which meant that in some cases the reported frequency of family walks increased. Children from higher SES schools reported awareness of safety, transport, and location (83%) as enabling factors for physical activity participation. Children specifically highlighted swimming as a popular activity (n = 44), and some reported that they had greater motivation to participate in this activity as it represented an opportunity for the family to spend time together.

Adults suggested a range of factors which enabled children's participation in physical activity (Additional file 3, Figure S3). Parents advocated children's engagement in physical activity in all weathers (n = 9), often describing their desires to play outdoors in snow for example. Dogs were identified as a key facilitator to physical activity, as they encouraged the family to go out for walks, regardless of the weather. Two adults (1 parent, 1 teacher) noted that, in their opinion, children of 9 and 10 years are at the age where they become conscious of their health and their body. Parents (n = 7) identified family holidays as an opportunity for the whole family to participate in a range of physical activities, overcoming, barriers such as parental social physique anxiety. Moreover, a number of families lived in quiet residential areas with low traffic, whereby parents felt it was activity promoting (n = 7) and relatively safe (n = 4) for their child to play outside. In some cases, teachers explained that children were occupying teaching roles with younger pupils by acting as play leaders, which was indicative of the children's physical activity self-efficacy and appreciation of the importance of physical activity. The adults also highlighted how facilities and equipment (n = 25), encouragement (n = 15), and transportation (n = 8) facilitated children's physical activity participation.

### Knowledge of physical activity and health

Data on children's knowledge (Additional file 4, Figure S4) revealed that physical activity was perceived most frequently by the participants as sport (n = 20) or exercise (n = 11). Some children (n = 14) correctly identified examples of physical activity, with few (n = 2) demonstrating limited knowledge. Children's knowledge of physical activity was tentative yet health knowledge was comfortably displayed (n = 35). Interestingly, of those who identified knowledge of the impact of low levels of physical activity, 80% were those with low SES backgrounds.

Data revealed (Additional file 5, Figure S5) that adult's demonstrated knowledge of the impact of regular physical activity (n = 23), and health (n = 9), though only one participant specifically distinguished between physical activity, sport and exercise:

"...it's not always about football and netball which seems to be the usual school activities, you know, there are different ways of being active." (F13)

The views indicated that parents, regardless of SES, understood the importance of their children being physically active.

## Discussion

The first aim of this study was to elicit the views of primary school children, their parents, and teachers in relation to their knowledge, behaviours, and perceptions towards physical activity, and to examine the perceived benefits and barriers to participation. This builds on previous research by using a new qualitative technique to inform the development of an intervention that will largely be delivered through schools, but will require family support to deliver on the objectives. The use of an emerging qualitative methodology enabled a comprehensive review of a large data set in conjunction with an established theoretical model. Pen profiles allowed a 'reader-friendly' representation of a quantitatively based analysis procedure therefore eliminating the likelihood of data, and hence key emergent themes, being skewed by dominating participants, whose views may be of the minority. This analysis technique therefore advances previous qualitative research studies by providing a basis of organising and representing key emergent themes. The second aim of the study was to use these formative data to inform the design of a tailored population-based physical activity intervention programme with the aim of enabling primary school children to develop healthy physical activity behaviours and make more informed lifestyle choices.

The data revealed a range of health knowledge in children and adults [17,37], but also identified lack of physical activity identification, which is contradictory to previous research [12]. Participants had a good understanding of the relationship between physical activity and health, although contrary to a previous study [12], some children demonstrated a limited understanding of what constitutes physical activity. Parents' views indicated that they understood the importance of their child being physically active, regardless of SES, which is consistent with more recent research [17]. Despite high levels of child and parent knowledge about the importance of physical activity engagement, this knowledge did not appear to always translate into actual physical activity behaviours. These results suggest that enhancing family based education on what constitutes physical activity, and how it can be incorporated into familial daily lifestyles, should be the focus of tailored interventions.

Fun, enjoyment, and social support were important predictors of physical activity participation and non-participation. Children see enjoyment and peer interaction as reasons to be physically active [11], prompting the development of interventions that maximise the fun and enjoyable aspects of physical activity [14]. Children from lower SES schools demonstrated autonomy over their physical activity through activities such as devising 'made up' games. When autonomy is conceptualised as choice as in Self-Determination Theory [38], then increased choice in active behaviours might have been expected to be demonstrated more by children from the higher SES schools. These children may, theoretically, have more opportunities for participation related to parental income, leisure time, and the value placed on active lifestyles [8]. Nonetheless, it could be drawn from these data that the lower socioeconomic areas are linked with more opportunity due to unsupervised play; parents from higher SES backgrounds advocated more sense of accompanying their children to the park, for example. Further, it may also be proposed that those children with lesser access to organized physical activity may have to rely on their imagination to devise games. Interestingly, teachers in a high SES school suggested that lack of structure during school playtime (i.e., recess) compromised participation in physical activity, contrary to reported literature conveying that the interventionist approach may have limited effects on physical activity and play behaviour [39,40]. Perhaps this reinforces why low SES school children expressed a sense of choice at playtime. Given that children with a sense of autonomy participated in regular physical activity, children's physical activity could be facilitated with a greater choice and variety of activities/opportunities [41,42]; thus, part of the intervention could provide suggestions for inexpensive and fun activities to do alongside family members.

Children reported participating in a variety of structured sports, such as organized football and swimming lessons. This supports previous research [12,17,43], though several barriers to physical activity engagement were also identified across all group interviews. The barriers elicited by children and parents were generally consistent with those presented in previous studies [17,44,45], with parents perceived by the children to be the biggest barriers to their physical activity participation (37%), regardless of SES or gender. Teachers also conveyed experiences of parents acting as barriers to their children's health and physical activity participation. For both parents and teachers, safety concerns were a significant perceived barrier to children's physical activity participation, particularly in relation to adverse weather and proximity of activity to busy roads, both of which were associated with restrictions on children's play [46-48]. While some children and adults reported weather preventing them from engaging in physical activity, supporting previous research [49,50], others noted that weather was not only perceived as less restrictive, but provided extra opportunities for participation. For example, both children and parents conveyed that snow provided opportunity for family physical activity:

"cause it snowed a lot over the winter, me and my friend [*name*], we were playing out like every single day making snow forts and having snowball fights." (B15)

However, it is noteworthy that snow in this north-west England Borough is infrequent; therefore it is more likely that it is the novelty which increases physical activity. Children and parents both identified that high levels of sedentary screen time (i.e., television and video-games) negatively impacted on physical activity [17], suggesting that the range of sedentary behaviours available may be more reinforcing than physical activity even when physically active alternatives are available [51]. Nonetheless, teachers did not advocate the negative association between screen time and physical activity, perhaps because they associate it with positive learning outcomes. Other barriers identified mainly by lower SES families included lack of money and transportation, both of which are consistent with previous research [12,17].

Despite similarities between enabling factors identified by adults and children, parents in particular perceived holidays as an opportunity for family based physical activity, perhaps as a result of overcoming time barriers associated with work and school commitments [37], thus allowing focus on leisure. Children, parents and teachers all reported that peers as well as families were major influences on children's physical activity participation [12,52], and dog ownership often led to increased frequencies of family walks [53]. Parental influences were thought to operate primarily through providing support and encouragement [11,54], but also through role modelling and providing opportunities for activity, which together influence children's learning, how children respond to the external environment, and what children expect of themselves [55]. Peer influences were seen as supportive by children, but as role models by teachers. Paradoxically, parents were both significant barriers (i.e., 'grounding') and enablers (i.e., encouraging) to children's physical activity participation, indicating that parents effectively have the greatest influence over their children's involvement in physical activity with the ability to both facilitate and impede participation [56]. Families, therefore, play a powerful and important role in promoting health-enhancing behaviours, thus involving parents and the whole family appears fundamental to approaches attempting to increase children's physical activity levels. Moreover, this approach should help overcome any potential conflicting messages between school and home-life.

In agreement with Power et al. [17] parents and teachers believed that schools were influential contexts for children's physical activity participation by offering various structured and unstructured opportunities for physically active pursuits. It is therefore important that the key features of the intervention are structured around both parents and schools. Further, within the intervention children need to receive support from teachers and parents in order to increase their perceptions of competence, self-efficacy and enjoyment [57].

The use of comprehensive formative research enabled depth of data to be gathered in a relatively short period of time. These findings will specifically be used to devise and implement an intervention for this population. A major strength of the study is not only supporting new methodologies within qualitative research, but advancing previous research utilising pen profiles [31] through the use of triangulating data between groups (i.e., children, parents and teachers). This research advances previous qualitative formative studies through the use of a large sample size. Other methodological strengths are the inclusion of both high and low socioeconomic backgrounds and the triangulation consensus of data between authors providing credibility, transferability, and dependability. Indeed, group interviews with children allowed an insight into their thoughts, beliefs and experiences towards physical activity, respecting the expert knowledge of the participant [58]. Moreover, triangulation between children's and parents and teachers decreased the risk of misinterpreted views and therefore potentially inaccurate data. There may be a risk that the data were influenced by sampling bias, though it is noteworthy that the majority of children (63%) in every school consented to take part.

## Conclusions

Group interviews revealed consistent themes between the socioeconomic groups, and gender for knowledge, behaviours, and perceptions towards physical activity. Aspects of the intervention can be modified depending on local need and resources, based on these findings. The results of this formative research will be used to inform the content and delivery of the physical activity component of the CHANGE! (Children's Health, Activity, and Nutrition: Get Educated!) health education intervention.

## Competing interests

The authors declare that they have no competing interests.

## Authors' contributions

KM participated in the design of the study, carried out the interviews, performed the analyses and drafted the manuscript. NR informed the analyses and helped to draft the manuscript. ZK acted as an expert in the analyses, triangulated the data and helped to draft the manuscript. SF conceived the study, participated in its design and coordination, and helped to draft the manuscript. All authors read and approved the final manuscript.

## Pre-publication history

The pre-publication history for this paper can be accessed here:

http://www.biomedcentral.com/1471-2458/11/831/prepub

## Supplementary Material

Additional file 1 **Children's Reinforcing Factors. Contains Figure S1 - A pen profile showing children's reinforcing factors**. B = Boy. G = Girl. Click here for file

Additional file 2 **Children's Enabling Factors. Contains Figure S2 - A pen profile showing children's enabling factors**. B = Boy. G = Girl. Click here for file

Additional file 3 **Adults' Perceived Enabling Factors to Children's Physical Activity. Contains Figure S3 - A pen profile showing adults perceived enabling factors to children's physical activity**. F = Female. Click here for file

Additional file 4 **Children's Knowledge of Physical Activity and Health. Contains Figure S4 - A pen profile showing children's knowledge of physical activity and health**. B = Boy. G = Girl. Click here for file

Additional file 5 **Adults' Knowledge of Physical Activity and Health. Contains Figure S5 - A pen profile showing adults' knowledge of physical activity and health**. F = Female. Click here for file
