# Supplementary material for: Using formative research to develop CHANGE!: a curriculum-based physical activity promoting intervention
Source: BMC Public Health. 2011 Oct 27;11:831. doi: 10.1186/1471-2458-11-831 (PMC3214189; doi:10.1186/1471-2458-11-831)
Supplement: Additional file 1 — Children's Reinforcing Factors. Contains Figure S1 - A pen profile showing children's reinforcing factors. B = Boy. G = Girl. [file 1471-2458-11-831-S1.DOC]

**Reinforcing**

**Parents n=25** ‘My Mum and Dad come and watch me play football...it just feels nice when someone’s watching ya’ B4

**Family n=50** ‘My family generally support me in what I want to do...so I think that will really help’ G36

**Peers n =23** ‘I would tell my friends and then some of them will be really supportive and like try to help me to reach the goal’ G14

**Coaches/Teachers n = 12** ‘Our teacher, Miss Bentley, she gives like us points because you are passing the ball to girls and it’s not just boys who are keeping the ball to themselves’ B10
