# Supplementary material for: Using formative research to develop CHANGE!: a curriculum-based physical activity promoting intervention
Source: BMC Public Health. 2011 Oct 27;11:831. doi: 10.1186/1471-2458-11-831 (PMC3214189; doi:10.1186/1471-2458-11-831)
Supplement: Additional file 2 — Children's Enabling Factors. Contains Figure S2 - A pen profile showing children's enabling factors. B = Boy. G = Girl. [file 1471-2458-11-831-S2.DOC]

**Weather n=17** ‘I like playing generally anywhere, out, outside me street, even in the snow. I run about chucking snowballs at everybody!’ B7

**Enabling**

**Safety n=2** ‘When I’m out she rings me up and she knows that I’m safe’ B11

**Dogs n=11** ‘Cause we have a dog it’s quite easy cause he needs to go out it kinds of makes us go out and take him for a walk and get some exercise’ G16

**Location n=17** ‘Me and Georgina live on the same like street and it’s like a cul de sac so not many cars go so we play out quite a lot’ G18

**Transport n=1** ‘I don't think that where you live really stops you cause you can get in your car with what your taking and go’ B10

**Facilities and Equipment n=58 ‘**You give the key ring in, play with what you want and when you’ve got bored with it or when it’s the end of break, you give the equipment back in and they give you the key ring back’ B21
