# Supplementary material for: Using formative research to develop CHANGE!: a curriculum-based physical activity promoting intervention
Source: BMC Public Health. 2011 Oct 27;11:831. doi: 10.1186/1471-2458-11-831 (PMC3214189; doi:10.1186/1471-2458-11-831)
Supplement: Additional file 3 — Adults' Perceived Enabling Factors to Children's Physical Activity. Contains Figure S3 - A pen profile showing adults perceived enabling factors to children's physical activity. F = Female. [file 1471-2458-11-831-S3.DOC]

**Weather n=9** ‘The fortnight we had the snow we went sledging every day’ F28

**Enabling**

**Safety n=4** ‘We live in quiet streets. You can go out and it is relatively safe’ F23

**Dogs n=9** ‘We’ve bought the puppy so that we will go out cause we’ve no option and we’ll go out as a family’ F10

**Location n=7** ‘We live in a cul de sac with a huge drive and they play out there’ F2

**Transport n=8** ‘They can catch the bus to Horwich so they’ve started catching the bus and going swimming in Horwich with their friends’ F18

**Facilities and Equipment n=25 ‘**It’s getting them interested at a young age, their bikes, roller-skating, getting out walking’ F19

**Health Conscious n=2** ‘I think they’re at that age as well where they understand more about, they’re becoming more aware of their body, how they look, em, you know, how, what they eat and their size’ F36

**Holiday n=7** ‘When we go on holiday and we go to parks, we spend all day walking’ F2

**Encouragement n=15** ‘Encourage them with clubs, exercise, football, dancing... activities that are energetic and hopefully they will carry them on’ F25
