# Supplementary material for: Using formative research to develop CHANGE!: a curriculum-based physical activity promoting intervention
Source: BMC Public Health. 2011 Oct 27;11:831. doi: 10.1186/1471-2458-11-831 (PMC3214189; doi:10.1186/1471-2458-11-831)
Supplement: Additional file 4 — Children's Knowledge of Physical Activity and Health. Contains Figure S4 - A pen profile showing children's knowledge of physical activity and health. B = Boy. G = Girl. [file 1471-2458-11-831-S4.DOC]

**Knowledge of Physical Activity & Health**

**Physical Activity**

**Impact of Physical Activity n=5** ‘It can affect your whole body and your whole life’ B10

**Health n=35** ‘It means like staying physically active and like eating healthily’ G18

**Physical Activity +ve n=14** ‘I jump on my bed’ G9 ‘Activities that you move around’ B18

**Physical Activity -ve n=2** ‘Is that exercise when you swing that ball around your foot?’ G7

**Exercise n=11** ‘Exercising and getting fit’ B22

**Sport n=20** ‘Sports and things like that’ B13
