# Supplementary material for: Using formative research to develop CHANGE!: a curriculum-based physical activity promoting intervention
Source: BMC Public Health. 2011 Oct 27;11:831. doi: 10.1186/1471-2458-11-831 (PMC3214189; doi:10.1186/1471-2458-11-831)
Supplement: Additional file 5 — Adults' Knowledge of Physical Activity and Health. Contains Figure S5 - A pen profile showing adults' knowledge of physical activity and health. F = Female. [file 1471-2458-11-831-S5.DOC]

**Knowledge of Physical Activity & Health**

**Impact of Physical Activity n=23**

‘I've given my kids the chance to do sport because I think it will benefit them no end through their lifetime’ F1

**Health n=9**

‘We’ve had heart problems on both sides of the family so you've got to get it before it gets too bad’ F2

**Physical Activity n=1**

It’s not always about football and netball which seems to be the usual school activities, you know, there are different ways of being active’ F13
